# Comparative microRNA profiling of sporadic and BRCA1 associated basal-like breast cancers

**DOI:** 10.1186/s12885-015-1522-4

**Published:** 2015-07-08

**Authors:** Max Yan, Kristy Shield-Artin, David Byrne, Siddhartha Deb, Nic Waddell, kConFab Investigators, kConFab, Izhak Haviv, Stephen B Fox

**Affiliations:** 1 Department of Anatomical Pathology, Prince of Wales Hospital, School of Medical Sciences, University of New South Wales, Randwick, 2031 Australia; 2 Baker IDI Heart and Diabetes Institute, Prahran, 3004 Australia; 3 Department of Pathology, Peter MacCallum Cancer Centre, East Melbourne, 3002 Australia; 4 Queensland Centre for Medical Genomics, Institute for Molecular Bioscience, University of Queensland, St Lucia, Australia

**Keywords:** Breast cancer, microRNA, BRCA1, Basal-like

## Abstract

**Background:**

While a number of studies have examined miRNA profiles across the molecular subtypes of breast cancer, it is unclear whether BRCA1 basal-like cancers have a specific miRNA profile. This study aims to compare grade independent miRNA expression in luminal cancers, sporadic and BRCA1 basal-type breast cancers. It also aims to ascertain an immunohistochemical profile regulated by BRCA1 specific miRNAs for potential diagnostic use.

**Methods:**

miRNA expression was assessed in 11 BRCA1 basal, 16 sporadic basal, 17 luminal grade 3 cancers via microarrays. The expression of Cyclin D1, FOXP1, FIH-1, pan-ERβ, NRP1 and CD99, predicted to be regulated by BRCA1 specific miRNAs by computer prediction algorithms, was assessed via immunohistochemistry in a cohort of 35 BRCA1 and 52 sporadic basal-like cancers. Assessment of cyclin D1, FOXP1, NRP1 and CD99 expression was repeated on a validation cohort of 82 BRCA1 and 65 sporadic basal-like breast cancers.

**Results:**

Unsupervised clustering of basal cancers resulted in a “sporadic” cluster of 11 cancers, and a “BRCA1” cluster of 16 cancers, including a subgroup composed entirely of 10 BRCA1 cancers. Compared with sporadic basal cancers, BRCA1 cancers showed reduced positivity for proteins predicted to be regulated by miRNAs: FOXP1 (6/20[30 %] vs. 37/49[76 %], *p* < 0.001), cyclin D1 (8/22[36 %] vs. 30/46[65 %], *p* = 0.025), NRP1 (2/20[10 %] vs. 23/46[50 %], *p* = 0.002). This was confirmed in the validation cohort (all *p* < 0.001). Negative staining for 2 or more out of FOXP1, cyclin D1 and NRP1 predicts germline BRCA1 mutation with a sensitivity of 92 %, specificity of 44 %, positive predictive value of 38 % and a negative predictive value of 94 %.

**Conclusion:**

Sporadic and BRCA1 basal-like cancers have grade independent miRNA expression profiles. Furthermore miRNA driven differences in the expression of proteins in BRCA1 basal cancers may be detected via immunohistochemistry. These findings may have important diagnostic implications, as immunohistochemical assessment of basal cancers, in addition to the patient’s family and clinical history, may potentially identify patients who may benefit from BRCA1 gene testing.

**Electronic supplementary material:**

The online version of this article (doi:10.1186/s12885-015-1522-4) contains supplementary material, which is available to authorized users.

## Background

Basal-like breast cancers are a subset of breast cancers characterised by triple negativity for ER, PR and HER2, and the expression basal/myoepithelial markers such as CK5/6 [1], CK14 [2] and EGFR [3]. They comprise approximately 15 % of all breast cancers [1, 4], and are associated with a more aggressive behaviour and also lack available targeted therapy. It is estimated that 2 % of all breast cancers are directly attributable to inherited mutations in the breast cancer susceptibility gene BRCA1 [5]. A strong link exists between BRCA1 mutations and basal phenotype, with 80–90 % of BRCA1 cancers expressing this phenotype [6].

microRNAs are small non-coding RNAs of 20–27 nucleotides that suppress translation through imperfect base pairing with their target mRNAs [7]. It is estimated that ~20 % of mRNA targeted by miRNAs undergo RISC mediated cleavage [8], The remainder may undergo translational silencing without associated changes in mRNA expression [9–11]. Although a number of studies examined miRNA profiles across the molecular subtypes of breast cancer [12–16], it is unclear whether the basal-like miRNA profiles obtained were independent of tumour proliferation and differentiation as defined by grade. The aim of this study is to: 1) derive a basal type miRNA signature that is independent of grade, 2) compare miRNA expression between sporadic and BRCA1 basal cancers to derive a BRCA1 basal signature using formalin fixed paraffin embedded (FFPE) tissue and 3) interrogate via immunohistochemistry the expression of proteins, predicted by computer algorithms, to be regulated by BRCA1 basal specific miRNAs. Identification of a basal type miRNA signature will aid in the targeting of specific miRNAs for further investigation. This is particularly important in view of the limited therapeutic options available for this particularly aggressive variant of breast cancer. It is has been shown that due to their fragment length, miRNA integrity does not appear to be affected storage as archival FFPE tissue [17]. Generation of a “BRCA1 basal” miRNA and immunohistochemical profile in FFPE tissue may identify patients with basal type cancers who will require BRCA1 genetic testing.

## Methods

### Samples for miRNA analysis

Forty-four primary grade III breast cancer (11 BRCA1 basal, 16 sporadic basal, 17 luminal) and 13 normal breast FFPE (formalin fixed, paraffin embedded) specimens were collected for the study. Definitions of intrinsic subtypes were based on ER, HER2 *in situ* hybridization, EGFR and CK5/6 staining, as per Nielson et al. [3]: Basal cancers (ER negative, HER2 negative, CK5/6 and/or EGFR positive), Luminal cancers (ER positive, HER2 negative). Basal cancers from patients with documented BRCA1 mutations were sourced from kConFab (www.kconfab.org), whereas normal breast tissue, sporadic basal and luminal cancers were collected from the Department of Pathology, Peter MacCallum Cancer Centre and the Victorian Cancer Biobank. Patients with sporadic basal cancers did not have a significant family history as defined by National Cancer Institute guidelines for BRCA1/BRCA2 mutation testing (www.cancer.gov). The clinico-pathological characteristics of the patients included in the study are listed in Additional file 1: Table S1. The study has ethics approval (Peter MacCallum Cancer Centre 09/36). For patients with sporadic cancers, due to the use of archival FFPE tissue, written informed consent was not required by the ethics committee. For BRCA1 patients, written informed consent was obtained as per kConFab (Kathleen Cuningham Foundation Consortium for research into Familial Breast cancer) biobank guidelines (www.kconfab.org). Three basal (HS578T, MDA-MB-231, MDA-MB-468, all with wild-type BRCA1) and two luminal (MDA-MB-453, MCF-7) breast cancer cell lines were also included in the study.

### RNA extraction

For primary tumours and normal breast tissue, 10 μm thick sections were cut from FFPE tissue blocks. The sections were dewaxed in xylene, placed through 100 % alcohol and allowed to dry. The samples were needle microdissected to ensure the proportion of tumour (or normal epithelium) was greater than 80 %, prior to placement into lysis buffer (Agencourt Formapure kit, Beckman Coulter, Beverly, MA, USA). Tissue was digested as per kit protocol (incubate at 70 ° C for 1 h, then add 20 μl of Proteinase K and incubate at 55 ° C for 1 h). Total RNA was extracted via a standard TRIZOL(Sigma)/chloroform protocol. For cell lines, total RNA was extracted using the total RNA protocol from the mirVana miRNA Isolation Kit (Ambion, TX, USA). All samples underwent DNase treatment with the Ambion DNA-free kit (Ambion, TX, USA).

### miRNA array

For each sample, 250 ng of total RNA was labelled and hybridized on Human v2 MicroRNA Expression BeadChips (Illumina, San Diego, CA, USA), according to the manufacturers recommendations (Illumina MicroRNA Expression Profiling Assay Guide). The layout of samples across the beadchips is shown in Additional file 1: Table S2. Sixty-nine samples (44 tumour, 13 normal, 7 controls and 5 cell lines) were hybridised on six beadchips across two separate runs: Run 1 (1 beadchip, 11 samples) and Run 2 (5 beadchips, 58 samples). The sample groups were randomised across the beadchips and also based on position within the beadchip. Controls were included for comparisons between the six beadchips and also between the two separate runs. The correlation of miRNA from control samples across the beadchips are outlined in Additional file 2: Figure S1.

The BeadChips were scanned with the Illumina iScan Reader. Data were imported into GenomeStudio (Illumina), from which raw data with background subtraction were exported to PARTEK Genomics Suite (St. Louis, Missouri, USA) for further analysis. Probes with a maximum intensity value of less than 150 units across all samples were excluded. Of the 1145 probes present on the array, 1037 were used for subsequent analyses. Raw probe intensities were shifted, such that the minimum probe intensity for each sample was equal to 1. All values were transformed by taking logs (base 2), followed by quantile normalisation [18]. Probe mapping for Illumina MicroRNA Expression v2 BeadChips was based on miRBase v.12.0.

Differential expression between groups was assessed using ANOVA, with inclusion of the Beadchip number as an independent variable to control for variations between Beadchips. A *p*-value of < 0.05, after Benjamini-Hochberg adjustment for multiple tests, was regarded as significant.

For each miRNA, the expression profiles were standardised to a mean of zero and a standard deviation of 1 prior to unsupervised hierarchical clustering. Clustering was performed using average linkage and Pearson correlation [12]. The full array data is available in GEO (Accession number: GSE61438).

### microRNA real time RT-PCR

Expression of microRNA (miRNA)s hsa-miR-374b, −190b, −198, −892a, −130b*, −218, −590-3p and −149 was assayed using real time RT-PCR. cDNA was reversed transcribed from total RNA samples using TaqMan MicroRNA assays and the TaqMan MicroRNA reverse transcription kit (Applied Biosystems). The cDNA was amplified using TaqMan microRNA Assay primers and the TaqMan Universal PCR Mastermix, according to the manufacturer’s instructions on the Roche Lightcycler 480. The relative miRNA expression levels were calculated by normalisation with RNU6B expression [19] using the second derivative (*Cp*) method [20]. Comparisons between groups were made using the un-paired *t*-test and correlations with array data were investigated using Pearson correlation on GraphPad Prism 5 (La Jolla, CA, USA).

### Prediction of miRNA targets

Predicted targets of miRNAs were identified via a union search of the two target prediction algorithms miRBase and TargetScan 5.1. Analysis of target protein expression by pSILAC (pulsed stable isotope labelling by amino acids in cell culture) suggests the specificity of these two algorithms are 44 and 61 % respectively [21]. Hence to improve specificity, only genes that are the predicted targets of three or more miRNAs differentially expressed between tumour groups was reported.

Ago2 immunoprecipitation studies by Karginov et al. have shown that approximately 20 % of mRNA targets undergo miRNA-induced cleavage. For the remainder of the targets (80 %), protein translation is suppressed without changes in mRNA levels [21, 22]. To identify the subset that undergoes miRNA-mediated cleavage, predicted targets derived from above were cross referenced with gene expression array data.

### Gene expression array

Gene expression data for 14 BRCA1 and 10 non-BRCA1 (5 BRCA2 and 5 BRCAX) basal cancers were derived from a cohort previously described by Waddell et al. (Data available on GEO, accession number GSE19177) [23]. RNA was extracted (Qiagen, Doncaster, VIC) from fresh frozen tissue and gene expression profiling was performed as per the manufacturer’s guidelines using 450 ng total RNA and Illumina Human-6 version 2 BeadChips containing 46,000 probes (Illumina Inc., San Diego, CA). Raw data were imported from Illumina Beadstudio v3.2 to PARTEK for further processing. Data were normalized with quantile normalisation, then filtered using an Illumina detection score of > 0.95 in at least one sample, which yielded 24,004 probes that were used in further analyses.

### Immunohistochemistry for miRNA targets

Tissue microarrays (TMAs) with single 1 mm cores were constructed. Cyclin D1, FOXP1, FIH-1, pan-ERβ, NRP1 and CD99 immunohistochemistry was performed on TMAs constructed from a cohort of 35 BRCA1 basal cancers from kConFab, and 52 sporadic basal cancers from the Instituti Ospitalieri di Cremona, Italy. Selection of antibodies was based on their previously described associations with BRCA1 status, wherever possible [24–28]. Immunohistochemistry for cyclin D1, FOXP1, NRP1 and CD99 was repeated on a second validation cohort composed of 82 BRCA1 basal cancers from kConFab, and 65 sporadic basal cancers from the Peter MacCallum Cancer Centre, Melbourne. TMA sections were cut from each block at 4 μm thick intervals, dewaxed, and placed through graded alcohol and placed into water. The antibody clones used and their titrations are listed in Additional file 1: Table S3. Antigen retrieval, incubation and visualisation for FIH and pan-ERβ were performed as per previous published studies [27, 29]. For NRP1 antigen retrieval was performed in a pressure cooker using high pH EnVision FLEX Target Retrieval Solution (Dako, Glostrup, Denmark) for 2 min. Antigen-antibody complex was detected using Envision FLEX system. FOXP1 and cyclin D1 staining was performed on the Ventana Benchmark® ULTRA system. Antigen retrieval was performed using Ventana ULTRA Cell Conditioner 1 and visualized with Ventana Ultraview Universal DAB. The intensity of staining was scored as negative = 0; weak staining = 1; moderate staining = 2; or strong staining = 3. The percentage of tumour cells stained in the given core scored as: 0 % = 0; 1–10 % = 1; 11–50 % = 2; 51–80 % = 3; 81–100 % = 4. The scores for both staining intensity and the percentage of positive tumour cells were added together to give a maximum score of 7. Comparisons between groups were based on a chi square (based on presence or absence of staining) and Mann-Whitney U tests (based on scores out of 7) performed on SPSS 16.0 (SPSS, IL, USA).

## Results

### Unsupervised hierarchical cluster analysis reveal distinct microRNA signatures among basal and luminal breast cancers

**Fig. 1:**
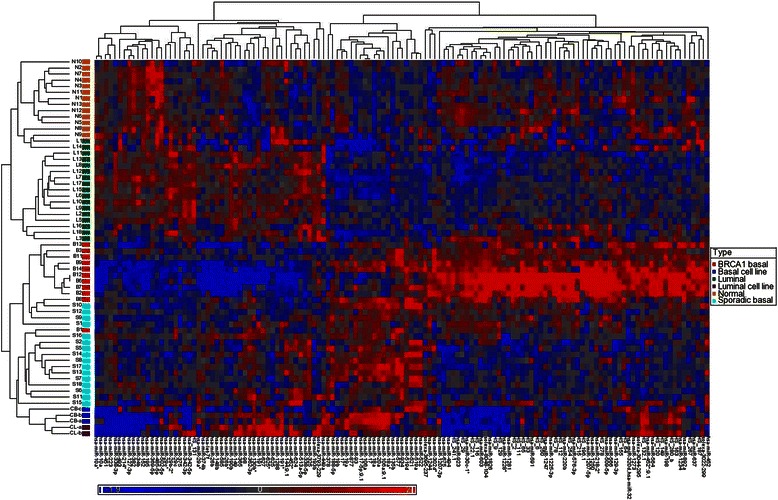
Unsupervised hierarchical cluster analysis over 133 classifying miRNAs (Pearson correlation, average linkage), all 62 samples

Unsupervised hierarchical clustering was performed on all 63 samples, based on the expression of 133 miRNAs. Selection of miRNAs was based on the top 75 miRNAs (based on fold change) differentiating between basal and luminal cancers and between BRCA1 and sporadic basal cancers (Fig. 1). To take into account the variation in probe intensity between the two runs (see above), standardisation for each miRNA to a mean of zero and a standard deviation of one was carried out separately for each run.

The analysis accurately separated normal tissue from breast cancer samples. It also separated basal from luminal cancers. There was some overlap (involving a cluster of 4 samples) between normal and luminal cancers. However it is noted that 3 of these 4 samples (normal N8, N9, luminal cancer L14), were from the smaller run of 11 samples. This overlap may be due to differences in hybridisation between the two different runs, despite an attempt to correct this by standardising the runs separately. Basal cell lines had a different miRNA profile compared to other primary basal cancer samples, and closely resembled the signature for luminal cell lines.

A number of miRNAs correlated with basal phenotype in the current and previous studies. miRNAs that were overexpressed in basal cancers across several studies (following adjustment of *p* values for multiple tests) include hsa-miR-17/*, 18a/b, 19a, 93, 106a/b, 135b and 142-5p. Similarly hsa-miR-29c/*, 109b, 342-3p/5p, 375 and hsa-let-7c were underexpressed in basal cancers (Table 1).

**Table 1:** Grade independent basal phenotype miRNA signature in common with other studies [12, 13, 49, 50]

	miRNA in common with other studies	Unadjusted *p* value	Adjusted *p* value	Fold change	Expression in basal vs. luminal
hsa-miR-149	[14, 16]	0.0246	0.2556	−2.21	Down
hsa-miR-29c	[14–16, 50, 51]	0.0315	0.2838	−1.22	Down
hsa-miR-29c*	[15, 16, 50]	0.0278	0.2679	−1.61	Down
hsa-miR-109b	[14, 50]	<0.0001	<0.0001*	−11.77	Down
hsa-miR-125a-5p	[14]	0.0203	0.2292	−1.13	Down
hsa-miR-136	[12]	0.0247	0.1120	−1.78	Down
hsa-miR-199a*:9.1	[12]	0.0138	0.0777	−1.47	Down
hsa-miR-342-3p	[14–16]	0 < 0.0001	0.0024*	−1.58	Down
hsa-miR-342-5p	[12–16, 49]	<0.0001	0.0001*	−1.97	Down
hsa-miR-375	[15, 16, 50]	0.0005	0.0177	−3.56	Down
hsa-let-7c	[12, 15]	0.0020	0.0232*	−1.26	Down
hsa-let-7f	[12]	0.0300	0.1284	−1.11	Down
hsa-let-7a	[12]	0.0467	0.1669	−1.05	Down
hsa-miR-17	[15, 16, 50]	<0.0001	0.0002*	1.74	Up
hsa-miR-17*	[14–16, 50]	0.0004	0.0162*	1.30	Up
hsa-miR-18a	[12–16, 32, 50, 52]	<0.0001	<0.0001*	5.22	Up
hsa-miR-18b	[14–16, 50]	0.0269	0.2676	1.89	Up
hsa-miR-19a	[14–16, 50]	0.0006	0.0191*	3.37	Up
hsa-miR-19b	[15, 16]	0.0005	0.0171*	1.90	Up
hsa-miR-93	[12, 13, 16]	0.0001	0.0032*	1.30	Up
hsa-miR-106a	[12, 13, 50]	<0.0001	0.0011*	1.87	Up
hsa-miR-106b	[12, 13, 15, 50]	0.0151	0.0814	1.59	Up
hsa-miR-135b	[12–16, 50]	<0.0001	0.0017*	4.44	Up
hsa-miR-142-5p	[12, 13]	0.0019	0.0229*	2.07	Up
hsa-miR-20a	[15, 16]	<0.0001	0.0001*	1.51	Up
hsa-miR-224	[14, 16]	0.0004	0.0162	4.75	Up
hsa-miR-455-5p	[14]	0.0159	0.1986	2.08	Up
hsa-miR-505	[50]	0.0030	0.0741	2.83	Up
hsa-miR-519a	[14]	<0.0001	0.0007*	9.05	Up
hsa-miR-521	[14]	<0.0001	<0.0001*	8.97	Up
hsa-miR-522	[14]	<0.0001	0.0004*	10.79	Up
hsa-miR-9	[14]	0.0069	0.1260	4.60	Up
hsa-miR-9*	[14, 16]	0.0322	0.2838	2.50	Up
hsa-miR-93	[15, 16]	0.0003	0.0142	1.32	Up

*significant *p* < 0.05 after adjustment for multiple tests

### Cluster analysis of basal cancers reveals miRNA signature enriched for BRCA1 cancers

**Fig. 2:**
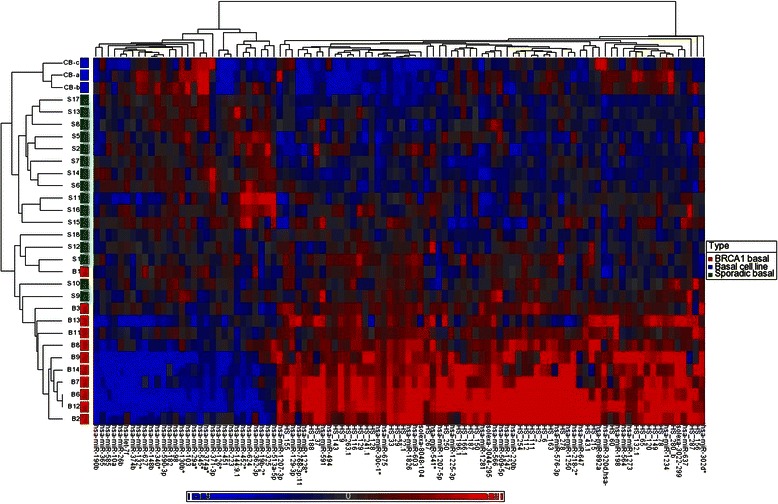
Unsupervised hierarchical cluster analysis, all basal cancers and basal cell lines, over 100 classifying miRNAs (Pearson correlation, average linkage)

A cluster analysis based on the top 100 miRNAs discriminating between BRCA1 and sporadic basal cancers was performed on all basal samples (including cell lines). This revealed two distinct signatures among the basal breast cancers (Fig. 2). A “BRCA1” rich cluster of 16 basal cancers, which included all 11 BRCA1 basal cancers plus 5 sporadic basal cancers, and a second “sporadic” basal cluster, composed of the remaining 11 sporadic basal cancers. Within the “BRCA1” cluster there was a subgroup composed entirely of 10 BRCA1 cancers. Basal cell lines, all with wild-type BRCA1, had a profile more closely resembling sporadic basal cancers rather than basal cancers with known BRCA1 mutations. miRNAs that are differentially expressed between BRCA1 and sporadic basal cancers, with a fold change of > 2.5, are listed in Table 2.

**Table 2:** miRNAs discriminating BRCA1 from sporadic basal cancers (fold change > 2.5, adjusted *p* < 0.05)

miRNA	Adjusted *p* value	Fold change	Expression in BRCA1 vs. basal	Cytoband	Correlation with previous BRCA1 CGH findings
hsa-miR-892a	0.0002	−7.98	Up	Xq27.3	
hsa-miR-1247	0.0015	−6.26	Up	14q32.31	
hsa-miR-129-3p	0.0001	−6.03	Up	11p11.2	
hsa-miR-494	0.0014	−5.84	Up	14q31	
hsa-miR-1273	0.0008	−5.43	Up	8q22.2	[53]
hsa-miR-198	0.0004	−5.34	Up	3q13.33	[54]
hsa-miR-30c-1*	<0.0001	−4.51	Up	1p34.2	
hsa-miR-1281	0.0001	−3.88	Up	22q13.2	
hsa-miR-220b	<0.0001	−3.76	Up	19p13.3	
hsa-miR-566	<0.0001	−3.75	Up	3p21.31	
hsa-miR-603	0.0003	−3.73	Up	10p12.1	[53, 55]
hsa-miR-675	0.0018	−3.53	Up	11p15.5	
hsa-miR-637	0.0012	−3.31	Up	19p13.3	
hsa-miR-576-3p	0.0014	−3.26	Up	4q25	
hsa-miR-638	0.0366	−3.04	Up	19p13.2	
hsa-miR-1826	<0.0001	−3.03	Up	16p11.2	[54]
hsa-miR-1268	0.0218	−2.96	Up	15q11.2	[53]
hsa-miR-1234	0.0076	−2.79	Up	8q24.3	[53, 56]
hsa-miR-1285	0.0428	−2.69	Up	7q21.2	
hsa-miR-509-5p	0.0043	−2.53	Up	Xq27.3	
hsa-miR-374b	0.0026	7.00	Down	Xq13.2	[53]
hsa-miR-590-3p	0.0101	5.25	Down	7q11.23	
hsa-miR-218	0.0264	4.02	Down	4p15.31	[56]
hsa-miR-335*	0.0428	3.82	Down	7q32.2	
hsa-miR-190b	0.0204	3.81	Down	1q21.3	
hsa-miR-96	0.0405	3.48	Down	7q32.2	
hsa-miR-454*	0.0405	3.21	Down	17q22	
hsa-miR-576-5p	0.0218	3.03	Down	4q25	
hsa-miR-340*	0.0111	3.01	Down	5q35.3	[53]
hsa-miR-29a*	0.0008	3.00	Down	7q32.3	
hsa-miR-148b	0.0014	2.90	Down	17q13.13	[56]
hsa-miR-130b*	0.0076	2.89	Down	22q11.21	
hsa-miR-149	0.0443	2.77	Down	17q37.3	
hsa-miR-10a	0.0076	2.71	Down	17q21.32	
hsa-miR-660	0.0428	2.56	Down	Xp11.23	

In order to assess the specificity of the miRNA array signals, seven miRNAs that discriminated between BRCA and sporadic basal cancers in the array were randomly chosen for measurement using reverse transcriptase real-time PCR. Differences between the two groups were validated for four of the seven miRNAs (mir-198, −374b, −218, −149) real-time PCR (*p* = 0.0015–0.0289) (Additional file 3: Figure S2), yielding a specificity of 57 %. For the 4 validated miRNAs, a moderate correlation was seen between data derived from the array and from RT-PCR (Pearson *r* = 0.503–0.672, all *p* < 0.05) (Additional file 4: Figure S3). There was a trend for lower mir-190b expression in BRCA1 cancers, although this was not statistically significant (*p* = 0.0914). No difference in mir-590-3p expression was seen between the two groups (*p* = 0.962). This may be due to reduced accuracy of measurement in the array at low concentrations, as reflected by the low concentrations for these 2 miRNAs (<2^−6^ relative to RNU6B) on RT-PCR. The concentration of mir-892a was below detection limit on RT-PCR (*Cp* > 35 cycles).

### Predicted targets of miRNA differentially expressed between BRCA1 and sporadic basal cancers

The predicted targets of the top 32 miRNAs differentially expressed between BRCA1 and basal cancers (excluding mir-892, −190b and 590-3p) were sought via a union search of miRBase and TargetScan. Predicted genes targeted by 3 or more miRNAs were retained for a subsequent analysis. This resulted in a list of 1218 genes predicted to be up (562) and down (656) regulated in BRCA1 basal cancers by miRNAs (Additional file 1: Table S4 and S5). Of these 1218 genes, there was overlap of 71 genes (5.8 %) between the two lists.

**Fig. 3:**
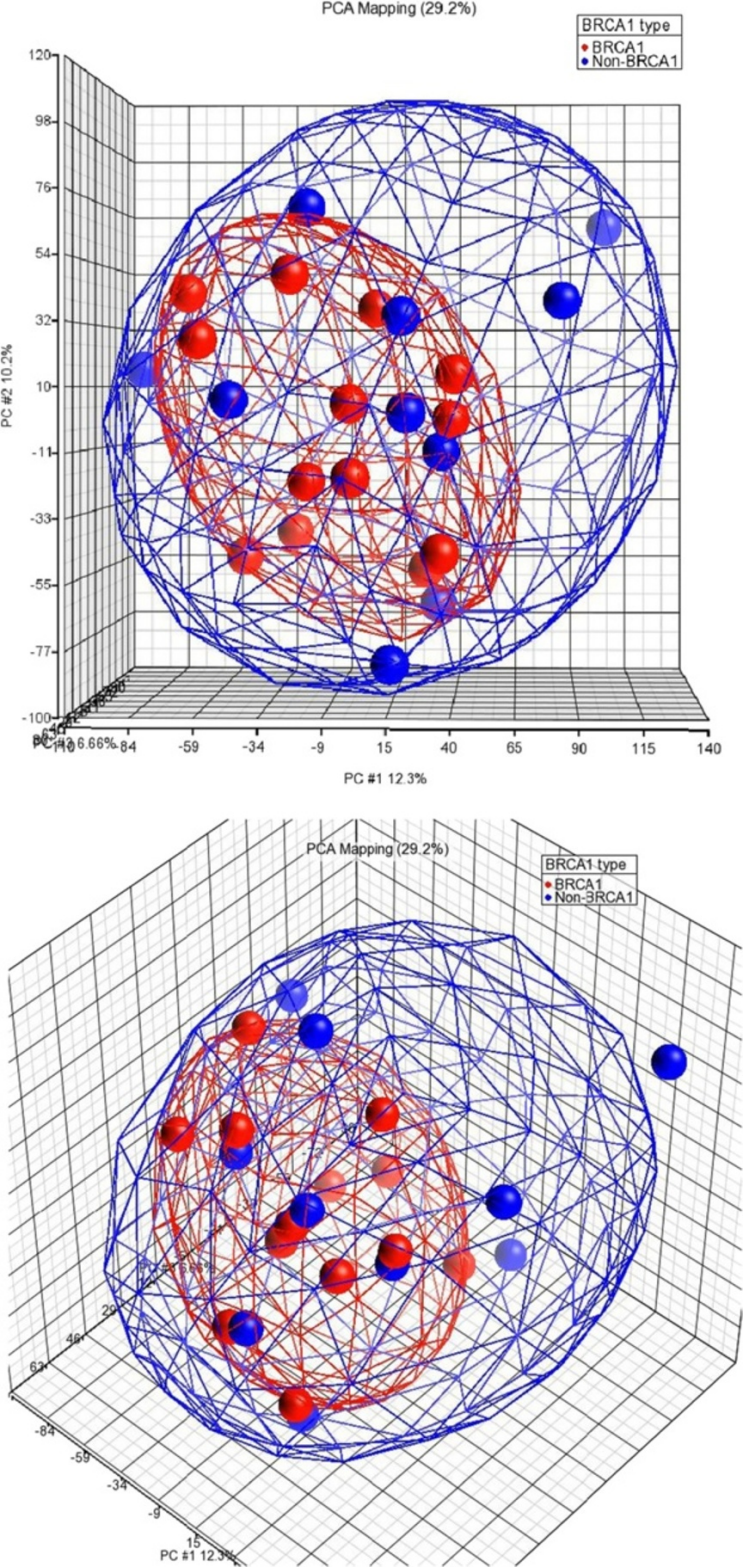
Three-dimensional PCA plot of gene expression array data for 14 BRCA1 and 10 non-BRCA1 basal cancers

The subset of predicted target mRNAs in BRCA1 and sporadic basal cancers, differentially expressed due to miRNA and RISC mediated cleavage was then investigated. Gene expression was compared between 14 BRCA1 basal and 10 non-BRCA1 basal cancers derived from Waddell et al.’s cohort. In a three-dimensional plot generated from an exploratory principle component analysis, the BRCA1 cancers formed a small cluster within a larger cluster incorporating all basal cancers (Fig. 3). Genes differentially expressed between BRCA1 and sporadic basal cancers were cross-matched with list of genes predicted to be targeted by differentially expressed miRNAs, to obtain a list of genes predicted to be regulated by RISC mediated cleavage (Additional file 1: Table S6).

### Immunohistochemical analysis of predicted miRNA targets in BRCA1 and sporadic basal cancers

The protein expression of four genes predicted to be down-regulated (cyclin D1, FOXP1, NRP1 and ERβ) and two predicted to be up-regulated (FIH-1 and CD99) by miRNA expression in BRCA1 vs. sporadic basal cancers were assessed via immunohistochemical staining on TMAs constructed from an cohort of 35 BRCA1 and 52 sporadic basal cancers. BRCA1 basal cancers, when compared to sporadic basal cancers showed reduced positivity for FOXP1 (6/20 [30 %] vs. 37/49 [76 %], *p* < 0.001), cyclin D1 (8/22 [36 %] vs. 30/46 [65 %], *p* = 0.025), NRP1 (2/20 [10 %] vs. 23/46 [50 %], *p* = 0.002) and CD99 (17/20 [85 %] vs. 7/19 [37 %], *p* = 0.002) (Table 3, Additional file 5: Figure S4). Differences in expression between the tumour groups were also significant when their scores (out of 7) were compared using a Mann-Whitney *U* test (all *p* ≤ 0.005). No differences in FIH-1 and ERβ expression were seen between the two groups (*p* > 0.05).

**Table 3:** miRNAs and expression of their predicted targets as assessed by immunohistochemistry (IPX) for the initial cohort of 35 BRCA1 and 52 sporadic basal cancers

miRNA	miRNA expression up or down in BRCA1 vs. sporadic cancers	Fold change miRNA	adjusted *p* value (BRCA1 vs. sporadic)	Predicted target gene	IPX expression up or down in BRCA1 vs. sporadic cancers	Positive IPX in BRCA1, *n* (%)	Positive IPX in sporadic, *n* (%)	*p* value (chi square) positive or negative on IPX	*p* value (Mann–Whitney) on IPX score out of 7
hsa-miR-509-5p	Up	2.53	0.004	FOXP1	Down	6 (30 %)	37 (76 %)	<0.001	<0.001
hsa-miR-1285	Up	2.68	0.042						
hsa-miR-1826	Up	3.02	<0.001						
hsa-miR-220b	Up	3.76	<0.001						
hsa-miR-1826	Up	3.02	<0.001	Cyclin D1	Down	8 (36 %)	30 (65 %)	0.025	0.004
hsa-miR-576-3p	Up	3.26	0.001						
hsa-miR-638	Up	3.04	0.036						
hsa-miR-30c-1*	Up	4.51	<0.001	NRP1	Down	2 (10 %)	23 (50 %)	0.002	0.003
hsa-miR-1285	Up	2.69	0.043						
hsa-miR-129-3p	Up	6.03	<0.001						
hsa-miR-29a*	Down	3.12	<0.001	CD99	Up	17 (85 %)	7 (37 %)	0.002	0.005
hsa-miR-130b*	Down	2.95	<0.006						
hsa-miR-132*	Down	2.48	0.024						
hsa-miR-340*	Down	3.09	0.028						

**Fig. 4:**
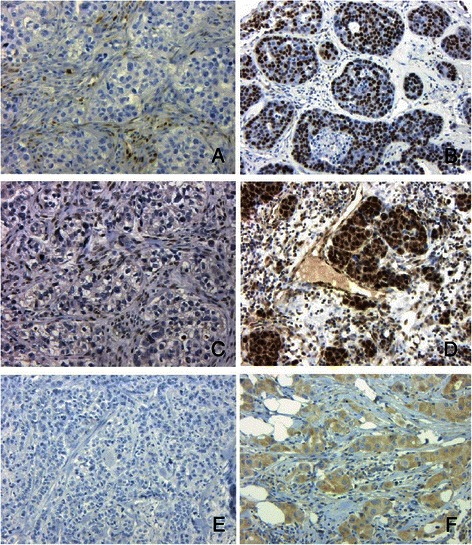
Negative immunoperoxidase staining for cyclin D1, FOXP1 and NRP1 in BRCA1 basal cancers (**a**, **b** and **e**). Positive staining in sporadic basal cancers for cyclin D1, FOXP1 and NRP1 (**b**, **d** and **f**). Note positive nuclear staining in stromal cells and negative staining in tumour cells for cyclin D1 (**a**) and FOXP1 (**c**). (x10, Haematoxylin counterstain)

Immunohistochemistry for FOXP1, cyclin D1, NRP1 and CD99 was repeated on a second validation cohort of 82 BRCA1 and 65 sporadic basal cancers. Statistically significant differences were confirmed for FOXP1, cyclin D1 and NRP1 (Pearson chi square all *p* < 0.001, Fig. 4 and Table 4). An opposite association for CD99 was observed with sporadic basal cancers showing increased expression compared to BRCA1 cancers (*p* = 0.024). Differences between the tumour groups were also observed when their scores (out of 7) were compared using a Mann-Whitney *U* test (all *p* < 0.001).

**Table 4:** Expression of FOXP1, cyclin D1 and NRP1, as assessed by immunohistochemistry (IPX) for the second validation cohort of 82 BRCA1 and 65 sporadic basal cancers

Predicted target gene	Positive IPX in BRCA1, *n* (%)	Positive IPX in sporadic, *n* (%)	*p* value (chi square) positive or negative on IPX	*p* value (Mann-Whitney) on IPX score out of 7
FOXP1	43 (52 %)	58 (94 %)	<0.001	<0.001
Cyclin D1	47 (57 %)	50 (89 %)	<0.001	<0.001
NRP1	33 (41 %)	42 (72 %)	<0.001	<0.001
CD99	23 (28 %)	26 (47 %)	0.024	0.012

Using negative staining for 2 or more out of 3 stains (FOXP1, cyclin D1 and NRP1) to predict germline BRCA1 mutation resulted in a sensitivity of 92 % and a specificity of 44 %. Assuming 1) the prevalence of BRCA1 in the breast cancer population is 2 % [5]; 2) 15 % of breast cancers have a basal phenotype [1, 4]; and 3) 69 % of all BRCA1 cancers have a basal phenotype [30], the positive predictive value (PPV) is estimated to be 38 % and the negative predictive value is estimated to be 94 %.

## Discussion

Interest in breast cancers with basal features began with Perou et al.’s paper based on cDNA arrays published in 2000 [31]. A number of studies assessing miRNA expression on basal cancers have been published since 2007 but none independent of grade. Thus, it has not been possible to date to reconcile any changes with veracity with regard to subtype or proliferation/differentiation, known drivers of mRNA and miRNA expression [12–16]. The current study, comparing 27 grade 3 basal-like with 17 luminal grade 3 cancers, confirms there are a number of miRNA differentially expressed between luminal and basal cancers, independent of grade, some of which including mir-17-92 cluster (mir-17, 17*, 18a, 19a/b, 20a and 106a), have been linked to basal-like phenotype in these previous studies [12, 14, 15, 32]. The expression of this cluster appears to be influenced by DNA copy number [15], and may be involved in the oncogenesis of basal-like cancers. These miRNAs promote tumour progression via: reduced stiffness of the extracellular matrix due to reduced expression of PTEN (mir-18a) [32], facilitation of cell migration and metastasis (mir-18b) [33], suppression of tumour suppressor genes ZBTB4 [34] and Rb(mir-106b) [35]. Loss of miRNAs may also be implicated in the activation of oncogenes, with loss of mir-375 and let-7a being implicated in epithelial-mesenchymal transition in breast cancer cells [36]. Our findings suggest these processes are specific to basal-like cancers, and are independent of tumour differentiation as reflected by the tumour grade.

Unsupervised clustering also suggests that basal-type cancers show significant heterogeneity in miRNA expression. This is in keeping with previous studies by Sotiriou et al. [37] and Kao et al. [38], who divided 26 basal cancers and 13 basal cell lines respectively into 2 subgroups, whereas Kreike et al. [39] divided 97 basal cancers into 5 subgroups. In this study, at one end of the spectrum, there is a subgroup composed entirely of 10 BRCA1 cancers, at the opposite end there is a cluster entirely composed of 11 sporadic cancers, which has a pattern of miRNA expression resembling *in vitro* basal cell lines. Thus, these data support our findings that BRCA1 cancers have a distinct miRNA signature and form a distinct subgroup within the basal cancers, characterised by the up-regulation of a number of miRNAs involved in regulation of the MAPK/ERK pathway (MPA3K2, MAP2K4, MAP4K4, PTPN2 [40], CHUK [41], PDGFRA [42], ERBB4, JAK3 [43]), and 2) histone modification (HDAC8, MYST2, MLL).

Of the six proteins that were investigated via immunohistochemistry, four (cyclin D1, FOXP1, NRP1 and CD99) showed reduced expression in BRCA1 cancers in the initial cohort with reduced expression for FOXP1, cyclin D1 and NRP1 subsequently confirmed in the validation cohort. Increased expression for CD99 in BRCA1 cancers was not reproduced in the validation cohort, where reduced expression was observed. While there were no accompanying changes in mRNA expression for these proteins, it is known that only ~ 20 % of mRNA targeted by miRNAs undergo RISC mediated cleavage. Hence it is likely that these three genes are part of the majority of genes that undergo translational silencing without an associated reduction in mRNA expression [21, 22].

Of these three genes, low cyclin D1 expression has been previously demonstrated in BRCA1 cancers [24, 25]. Reduced cyclin D1 expression in BRCA1 basal cancers may, in part, be due to the inhibition of translation mediated by mir-576-3p, mir-1826 and mir-638. Conversely, increased cyclin D1 expression in sporadic basal cancers may have important biological implications. For instance, direct targeting of cyclin D1 by NOTCH1 and 3 has been linked to cell cycle progression in basal cancers [44]. In addition, phosphorylation of BRCA1 by cyclin D1 has been shown to interfere with DNA dependent activities of BRCA1 [45]. Expression of NRP1 in sporadic cancers may have potentially important therapeutic applications, in view of the development of anti-NRP monoclonal antibodies and cell penetrating peptides [46].

Using negative staining for 2 or more out of 3 stains (FOXP1, cyclin D1 and NRP1) to predict germline BRCA1 mutation resulted in a positive predictive value (PPV) of 38 % and a negative predictive value of 94 %. While this PPV is relatively low due to the rarity of BRCA1 mutations in the population, its value is likely to improve in patients with a family history of breast cancer and/or early onset cancers. Nevertheless, our findings suggest immunohistochemistry for FOXP1/NRP1/cyclin D1 may be useful, in conjunction with family history, in selecting patients with basal cancers for BRCA1 screening.

## Conclusion

In summary, our study demonstrates basal-like cancers have a grade independent miRNA expression profile. Furthermore miRNA driven differences in the expression of proteins by BRCA1 vs. sporadic basal cancers may be detected via immunohistochemical staining of paraffin embedded tissue. These findings may have important diagnostic implications, as immunohistochemical assessment of basal cancers, in addition to the patient’s family and clinical history, may potentially identify patients who may benefit from BRCA1 gene testing. Lastly, there is evidence to suggest BRCA1 deficient cancers may be sensitive to PARP inhibitors [47]. Hence stratification of basal-like cancers, based on the “BRCA1-ness” of their miRNA signature, generated from archival FFPE tissue, may be highly relevant to clinical trials investigating targeted therapies, such as PARP inhibitors [48]. Validation of these findings in larger patient cohorts, however, will be required to assess the diagnostic utility of such an approach.

## Additional files

Additional file 1: Table S1. Clinico-pathological characteristics of tumour samples (all grade III) for miRNA analyses. **Table S2.** Layout of samples across 6 Illumina miRNA Beadchips. **Table S3.** Antibodies used for immunohistochemical staining of tissue microarrays. **Table S4.** Predicted genes up regulated in BRCA1 basal cancers, targeted by 3 or more miRNAs. **Table S5.** Predicted genes up regulated in sporadic basal cancers, targeted by 3 or more miRNAs. **Table S6.** Subset of predicted target mRNAs regulated by RISC mediated cleavage in BRCA1 and sporadic basal cancers.
Additional file 2: Figure S1. Correlation of control miRNA profiles across beadchips. The probe intensities for control samples were correlated across the 6 Illumina beadchips hybridised on 2 separate runs (Run 1: 1 beadchip with 11 samples, Run 2: 5 beadchips with 58 samples). Excellent correlations for raw miRNA probe intensities were obtained between control samples across the five beadchips hybridised on the same run (Run 2, Spearman *r* = 0.9631–0.9906, all *p* < 0.001) (Additional file 2: Figure S1a). For control samples hybridised on different runs, a weaker correlation was observed (Spearman *r* = 0.8139, *p* < 0.001) (Additional file 2: Figure S1b). Occasional probes were observed to have a much stronger signal in the smaller run 1 (4687786010_L) compared to run 2 (4726968002_E). A small improvement in the correlation between these two samples was observed following quantile normalisation and log 2 transformation (Pearson *r* = 0.8538, *p* < 0.001) (Additional file 2: Figure S1c). Additional file 2: Figure S1a. Scatterplot of raw miRNA probe intensities from control samples across 2 beadchips hybridised on the same run (Spearman *r* = 0.9906, *p* < 0.001); Additional file 2: Figure S1b. Scatterplot of raw miRNA probe intensities from control samples hybridised on separate runs (Spearman *r* = 0.8139, *p* < 0.001); Additional file 2: Figure S1c. Scatterplot of probe intensities of control samples (from Additional file 2: Figure S1b), hybridised on separate runs following quantile normalisation and log 2 transformation (Pearson *r* = 0.8538, *p* < 0.001).
Additional file 3: Figure S2. RT-PCR validation of mir-374b, mir-149, mir-218 and mir-198 in discriminating between BRCA1 and sporadic basal breast cancers (all *p* < 0.05). A trend was seen for mir-190b although this was not statistically significant (*p* = 0.0914). No differences in the expression of mir-590-3p were observed (*p* = 0.9623).
Additional file 4: Figure S3. Correlation between microarray and RT-PCR (relative to RNU6B) measurements for mir-198 (*r* = 0.672, *p* = 0.0023), mir-149 (*r* = 0.567, *p* = 0.0344), mir-218 (*r* = 0.521, *p* = 0.0385) and mir-374b (*r* = 0.503, *p* = 0.0335).
Additional file 5: Figure S4. Distribution of scores in BRCA1 and sporadic basal type cancers for cyclin D1 (*p* = 0.004), FOXP1 (*p* < 0.001) and NRP1 (*p* = 0.003).
