# Supplementary material for: Comparative microRNA profiling of sporadic and BRCA1 associated basal-like breast cancers
Source: BMC Cancer. 2015 Jul 8;15:506. doi: 10.1186/s12885-015-1522-4 (PMC4494690; doi:10.1186/s12885-015-1522-4)
Supplement: Additional file 1: Table S1. — Clinico-pathological characteristics of tumour samples (all grade III) for miRNA analyses. Table S2. Layout of samples across 6 Illumina miRNA Beadchips. Table S3. Antibodies used for immunohistochemical staining of tissue microarrays. Table S4. Predicted genes up regulated in BRCA1 basal cancers, targeted by 3 or more miRNAs. Table S5. Predicted genes up regulated in sporadic basal cancers, targeted by 3 or more miRNAs. Table S6. Subset of predicted target mRNAs regulated by RISC mediated cleavage in BRCA1 and sporadic basal cancers. [file 12885_2015_1522_MOESM1_ESM.docx]

**Supplementary table 1** Clinico-pathological characteristics of tumour samples (all grade III) for miRNA analyses

|  | **BRCA1 basal** | **Sporadic basal** | **Luminal** | **p value*** |
| --- | --- | --- | --- | --- |
| Median age (years) | 42.0 | 60.0 | 55.5 | 0.011 |
| Median size (mm) | 18 | 22 | 20 | 0.087 |
| Lymph node:  Negative  Positive  Unknown | 7 (78%)  2 (22%)  2 | 13 (82%)  2 (15%)  0 | 8 (47%)  9 (53%)  0 | 0.007 |

* ANOVA test for age and size, chi square test for lymph node status

**Supplementary table 2** Layout of samples across 6 Illumina miRNA Beadchips

| **Run number** | **Illumina chip ID** | **Chip position** | **Sample ID** | **Sample Group** | **Tissue** |
| --- | --- | --- | --- | --- | --- |
| 2 | 4687786009 | A | Control 1 | Luminal Cell Line | Cell line |
| 2 | 4687786009 | B | B1 | BRCA1 basal | FFPE |
| 2 | 4687786009 | C | L1 | Luminal | FFPE |
| 2 | 4687786009 | D | S1 | Sporadic basal | FFPE |
| 2 | 4687786009 | E | N1 | Normal | FFPE |
| 2 | 4687786009 | F | L10 | Luminal | FFPE |
| 2 | 4687786009 | G | S10 | Sporadic basal | FFPE |
| 2 | 4687786009 | I | CB-a | Basal Cell Line | Cell line |
| 2 | 4687786009 | J | S12 | Sporadic basal | FFPE |
| 2 | 4687786009 | K | L11 | Luminal | FFPE |
| 2 | 4687786009 | L | N11 | Normal | FFPE |
| 1 | 4687786010 | A | B8 | BRCA1 basal | FFPE |
| 1 | 4687786010 | B | L14 | Luminal | FFPE |
| 1 | 4687786010 | C | N10 | Normal | FFPE |
| 1 | 4687786010 | D | S11 | Sporadic basal | FFPE |
| 1 | 4687786010 | E | B9 | BRCA1 basal | FFPE |
| 1 | 4687786010 | F | L16 | Luminal | FFPE |
| 1 | 4687786010 | G | N8 | Normal | FFPE |
| 1 | 4687786010 | H | S16 | Sporadic basal | FFPE |
| 1 | 4687786010 | J | L8 | Luminal | FFPE |
| 1 | 4687786010 | K | N9 | Normal | FFPE |
| 1 | 4687786010 | L | Control 2 | Sporadic basal | FFPE |
| 2 | 4687786020 | A | L12 | Luminal | FFPE |
| 2 | 4687786020 | B | N12 | Normal | FFPE |
| 2 | 4687786020 | C | CL-a | Luminal Cell Line | Cell line |
| 2 | 4687786020 | D | B11 | BRCA1 basal | FFPE |
| 2 | 4687786020 | E | L13 | Luminal | FFPE |
| 2 | 4687786020 | F | S13 | Sporadic basal | FFPE |
| 2 | 4687786020 | G | Control 1 | Luminal Cell Line | Cell line |
| 2 | 4687786020 | H | L15 | Luminal | FFPE |
| 2 | 4687786020 | I | S14 | Sporadic basal | FFPE |
| 2 | 4687786020 | J | B12 | BRCA1 basal | FFPE |
| 2 | 4687786020 | K | N13 | Normal | FFPE |
| 2 | 4687786020 | L | S15 | Sporadic basal | FFPE |
| 2 | 4687786022 | A | B13 | BRCA1 basal | FFPE |
| 2 | 4687786022 | B | L17 | Luminal | FFPE |
| 2 | 4687786022 | C | S17 | Sporadic basal | FFPE |
| 2 | 4687786022 | D | N2 | Normal | FFPE |
| 2 | 4687786022 | E | Control 1 | Luminal Cell Line | Cell line |
| 2 | 4687786022 | F | S18 | Sporadic basal | FFPE |
| 2 | 4687786022 | G | B14 | BRCA1 basal | FFPE |
| 2 | 4687786022 | H | CB-b | Basal Cell Line | Cell line |
| 2 | 4687786022 | I | L18 | Luminal | FFPE |
| 2 | 4687786022 | J | CB-c | Basal Cell Line | Cell line |
| 2 | 4687786022 | K | S2 | Sporadic basal | FFPE |
| 2 | 4687786022 | L | L2 | Luminal | FFPE |
| 2 | 4726968002 | A | L3 | Luminal | FFPE |
| 2 | 4726968002 | B | N4 | Normal | FFPE |
| 2 | 4726968002 | C | B2 | BRCA1 basal | FFPE |
| 2 | 4726968002 | D | L5 | Luminal | FFPE |
| 2 | 4726968002 | E | Control 2 | Sporadic basal | FFPE |
| 2 | 4726968002 | F | N3 | Normal | FFPE |
| 2 | 4726968002 | G | Control 1 | Luminal Cell Line | Cell line |
| 2 | 4726968002 | H | N5 | Normal | FFPE |
| 2 | 4726968002 | I | B3 | BRCA1 basal | FFPE |
| 2 | 4726968002 | J | S5 | Sporadic basal | FFPE |
| 2 | 4726968002 | K | L6 | Luminal | FFPE |
| 2 | 4726968002 | L | S6 | Sporadic basal | FFPE |
| 2 | 4726968016 | A | S7 | Sporadic basal | FFPE |
| 2 | 4726968016 | B | B6 | BRCA1 basal | FFPE |
| 2 | 4726968016 | C | N6 | Normal | FFPE |
| 2 | 4726968016 | D | S8 | Sporadic basal | FFPE |
| 2 | 4726968016 | E | L7 | Luminal | FFPE |
| 2 | 4726968016 | F | B7 | BRCA1 basal | FFPE |
| 2 | 4726968016 | G | S9 | Sporadic basal | FFPE |
| 2 | 4726968016 | H | CL-b | Luminal Cell Line | Cell line |
| 2 | 4726968016 | I | Control 1 | Luminal Cell Line | Cell line |
| 2 | 4726968016 | J | L9 | Luminal | FFPE |
| 2 | 4726968016 | K | N7 | Normal | FFPE |

**Supplementary table 3** Antibodies used for immunohistochemical staining of tissue microarrays

| **Antibody** | **Clone** | **Source** | **Retrieval time (min)** | **Retrieval temp (^o^C)** | **Dilution** | **Incubation time (minutes)** | **Incubation temp (^o^C)** |
| --- | --- | --- | --- | --- | --- | --- | --- |
| Cyclin D1 | Rabbit monoclonal SP4 | Thermo scientific, Fremont, CA, USA | 36 | 95 | 1:50 | 32 | Room temp |
| FOXP1 | Rabbit polyclonal | Cell Marque, Rocklin, CA, USA | 36 | 95 | 1:50 | 32 | Room temp |
| FIH-1 | Mouse monoclonal 162c | Nuffield Department, John Radcliffe Hospital, Oxford, UK ([24](#_ENREF_24)) | Nil | N/A | Neat | 60 | Room temp |
| pan-ERβ | Mouse monoclonal 14C8 | Abcam | 20 | 100 | 1:200 | overnight | 4 |
| NRP1 | Rabbit monoclonal | Invitrogen, Camarillo, CA, USA | 2 | Pressure cooker | 1:100 | overnight | 4 |
| CD99 | Mouse monoclonal  12E7 | Dako | 52 | 95 | 1:200 | 32 | Room temp |

**Supplementary table 4** Predicted genes up regulated in BRCA1 basal cancers, targeted by 3 or more miRNAs

| **7 mirs** | EPHA8 | UNKL | CD99 | GADD45A | MRPL43 | RABGAP1L | TMSB10 |
| --- | --- | --- | --- | --- | --- | --- | --- |
| CLCN5 | EXOC5 | VAMP8 | CDY1 | GAPVD1 | MRPL9 | RAP1GAP | TNC |
| MECP2 | FBXO36 | VPS13A | CECR6 | GDF11 | MRPS16 | RAPH1 | TNRC6A |
| **6 mirs** | FOXP2 | WISP3 | CEP63 | GDF2 | MRPS21 | RBM14 | TPM1 |
| AAK1 | FRS2 | ZFYVE19 | CER1 | GDPD1 | MSH2 | RBM27 | TRAPPC6A |
| BAT4 | FZD5 | ZNF23 | CFLAR | GGN | MSN | RCC2 | TREX2 |
| BCL2L11 | GFOD2 | **3 mirs** | CGRRF1 | GIMAP2 | MTF1 | RCOR1 | TRIM7 |
| CCT3 | GNG11 | AASS | CHCHD2 | GIYD2 | MTMR14 | RCSD1 | TRIM9 |
| TANC2 | GRIA3 | ABCA1 | CHCHD9 | GJA4 | MTSS1 | REPS2 | TRPM6 |
| **5 mirs** | HAS3 | ABCA2 | CHUK | GOLGA3 | MUS81 | RGS1 | TRRAP |
| AFF4 | HBE1 | ABLIM1 | CLCN6 | GON4L | MYBPH | RICTOR | TSGA13 |
| C10orf119 | HIPK2 | ACAD11 | CLK2 | GPR172A | MYST2 | RIMS4 | TSPAN1 |
| C9orf86 | HOXB3 | ACPP | CLOCK | GPR85 | NCOA6 | RNF139 | TSPAN14 |
| CBWD3 | HP1BP3 | ADAMTS6 | CNOT6 | GPR98 | NDST1 | RNF219 | TTK |
| CRTAC1 | IGF1 | ADCK2 | CNOT6L | GRM1 | NDUFS2 | RNF38 | TUBAL3 |
| EIF2C1 | IL16 | ADCY1 | COL27A1 | GZMM | NEUROD1 | RNF41 | TUBB6 |
| ESRRG | INHBB | AFF2 | COL2A1 | HB25 | NFATC3 | RNF44 | TXNDC1 |
| FAT3 | INTS6 | AGPAT3 | COMMD6 | hCG_18385 | NLRP11 | ROGDI | UBAP2 |
| FRMD4A | KIAA1107 | AGPAT5 | COQ10B | HDAC8 | NPHS1 | ROM1 | UBE2H |
| FUT9 | LMBR1L | AGXT2L1 | CORT | HEATR5B | NPTX1 | RTN4 | UBFD1 |
| HNF4G | LMBRD1 | AHI1 | CPEB4 | HIF1AN | NPTX2 | SAMD13 | UBXD8 |
| HSPA4L | LRP2 | AHR | CPSF6 | HIF3A | NRP1 | SASH1 | UGP2 |
| KLF3 | MARK1 | AK2 | CPT2 | HLA-E | NT5DC1 | SATB1 | UMODL1 |
| LSM14B | MBNL1 | ALG12 | CRTC2 | HLF | NUDT5 | SCAMP2 | USP15 |
| MAK10 | MDGA2 | ANK1 | CRTC3 | HOXA10 | NUFIP2 | SCFD1 | USP32 |
| MMP10 | MED1 | ANKRD1 | CSMD2 | HRBL | NUP50 | SCN2A | USP33 |
| MTMR12 | MFSD9 | ANKRD7 | CTGLF1 | HS2ST1 | OCIAD1 | SEC22C | USP34 |
| NPSR1 | MYLK | ANKS1B | CTPS | HSD17B7 | ODZ4 | SEMA5A | USP45 |
| PCYT1B | MYO16 | ANKS6 | CTSD | HSPC159 | OR13C8 | SENP3 | USP6 |
| PHC3 | MYT1L | ANKZF1 | CXorf28 | HTATIP2 | OR51I2 | SERBP1 | VLDLR |
| PKN2 | NFAT5 | ANTXR2 | CYB5A | IFNK | P2RY6 | SERINC3 | VPS37A |
| PRRT3 | NFIX | ANXA13 | CYHR1 | IFRD2 | PAPPA | SESN3 | VTI1B |
| SNTB2 | NOVA1 | AP2A1 | DAZAP1 | IL17RA | PAQR3 | SFRS2IP | WFDC8 |
| TGM1 | NP_061959.2 | AP3M1 | DCBLD1 | IL6 | PBX2 | SFRS8 | WIPF1 |
| TLE4 | OLFML2B | APOB | DCX | IPO9 | PCDHA1 | SHB | WIPI2 |
| TNXB | ONECUT2 | APOBEC3G | DDAH1 | ITFG1 | PCGF5 | SLC12A1 | XIAP |
| TTBK2 | OXSR1 | APOBEC3H | DDOST | ITGAD | PCMT1 | SLC12A5 | YWHAH |
| USP47 | PBXIP1 | AQP11 | DEPDC1 | ITPR1 | PCNX | SLC12A6 | ZBTB34 |
| WDFY4 | PEA15 | ARG1 | DGKH | JAK3 | PDE4B | SLC26A10 | ZBTB44 |
| **4 mirs** | PHF19 | ARHGAP21 | DLG2 | JHDM1D | PDGFRA | SLC26A5 | ZC3H6 |
| ABAT | PHF20 | ARHGAP22 | DLGAP2 | KATNA1 | PFKFB3 | SLC33A1 | ZDHHC17 |
| ACVR2B | PLSCR5 | ARHGAP29 | DMD | KCNA1 | PGGT1B | SLC39A5 | ZDHHC24 |
| ADRA1A | PMEPA1 | ARHGEF12 | DYNLT3 | KCNK15 | PHF12 | SLC39A6 | ZDHHC5 |
| ALDH4A1 | PNRC1 | ARPP-19 | DYRK1A | KCNMA1 | PHF15 | SLC5A10 | ZEB2 |
| ANKRD44 | PPARGC1A | ART3 | EBF1 | KCTD16 | PHF21A | SLC9A2 | ZFAT1 |
| ANKRD52 | PPIE | ASB4 | EBF3 | KIF27 | PIK3C2A | SMAD2 | ZNF205 |
| ANKRD56 | PTGER3 | ATP13A4 | EDEM3 | KRT20 | PIK3CA | SNHG5 | ZNF768 |
| ANTXR1 | PTPRK | ATP1A2 | EFHC1 | LAMA4 | PITRM1 | SNX18 |  |
| ANUBL1 | Q8IXV1 | ATP2A2 | EFNA4 | LARP5 | PLA2G4F | SNX4 |  |
| ARPM1 | RALGPS1 | ATP5F1 | EFNB2 | LECT1 | PLA2G5 | SOCS4 |  |
| ARSG | RASGRP2 | ATP6V1G3 | EGF | LEO1 | PLCG1 | SOCS6 |  |
| ATP8A1 | RBBP6 | ATXN1 | EGR3 | LMO7 | POLDIP2 | SPAST |  |
| B3GAT3 | RBM9 | ATXN7L1 | ELOVL5 | LMTK2 | POP4 | SPTB |  |
| BACH2 | RC3H1 | BAI3 | EMR1 | LOC255411 | POPDC2 | SRPK2 |  |
| BCOR | RFX4 | BAZ2B | ENTHD1 | LPAR1 | POU5F1P1 | SSX3 |  |
| BMPR1B | RHOBTB1 | BCAT1 | EPAS1 | LPHN1 | PPARD | ST7L |  |
| BRUNOL6 | RNF165 | BCL11A | EPN1 | LRMP | PPIL4 | ST8SIA1 |  |
| BZRAP1 | RPS29 | BET3L | ERBB4 | LRP6 | PPP1R12B | ST8SIA3 |  |
| C14orf121 | SEC61A2 | BMX | ERCC5 | LRRC58 | PPP1R12C | STAP1 |  |
| C18orf1 | SLC25A15 | BRPF3 | ETNK1 | LRRC7 | PRDM14 | STK19 |  |
| C5orf30 | SLC5A3 | BSDC1 | ETNK2 | LTBP1 | PRKCE | STK24 |  |
| C6orf213 | SMS | BTBD8 | F13A1 | LUZP1 | PRLR | STXBP5 |  |
| C9orf39 | SOBP | CABP1 | FAM13A1 | LYSMD3 | PRSS16 | SULT1A3 |  |
| CBWD6 | SOS1 | CACNA1C | FAM81A | MAD2L2 | PSMD5 | TACC1 |  |
| CCDC4 | SOX5 | CACNA1S | FAM83C | MAP2K4 | PSPC1 | TADA2L |  |
| CCDC6 | SP1 | CADM2 | FCHO2 | MAP3K2 | PTBP1 | TAF12 |  |
| CD72 | SPIRE1 | CALCR | FCRL2 | MAP4K4 | PTCHD2 | TBC1D22B |  |
| CDK6 | SRA1 | CALN1 | FGD2 | MCFD2 | PTGDS | TBL1X |  |
| CETN3 | SSX2IP | CAPS2 | FGFR2 | MED10 | PTPN2 | TBP |  |
| CNTNAP2 | ST3GAL4 | CAPZA2 | FKBP1A | MED12L | PTPRE | tcag7.1228 |  |
| COL16A1 | TAF4B | CASP10 | FMO6P | MEF2D | PURB | TCHP |  |
| CPN1 | TEAD1 | CBWD1 | FMR1 | METTL8 | PURG | TDRD7 |  |
| CREB3L2 | TM6SF1 | CBWD5 | FOXK2 | MGAT4A | PVRL1 | TEAD3 |  |
| CTDSPL | TMEM19 | CBX2 | FRAS1 | MITF | Q5JZ71 | THOP1 |  |
| CUGBP2 | TNIP2 | CCDC22 | FREM1 | MLANA | Q5T6R2 | TIMM44 |  |
| DMBT1 | TNRC5 | CCDC64 | FRY | MLL | Q6NZ63 | TMEFF1 |  |
| EBP | TNRC6B | CCL14 | FUT4 | MOBKL1A | Q6ZW55 | TMEM112B |  |
| ELAVL2 | TOMM70A | CCL18 | FXYD2 | MPDU1 | Q8IX75 | TMEM132B |  |
| ELL2 | TOX | CCNI | GAB2 | MPDZ | Q8N1L4 | TMEM170B |  |
| ENSA | TPP2 | CD244 | GABRA6 | MPP1 | Q96FU4 | TMEM187 |  |
| EPB41L1 | TRO | CD58 | GABRE | MRPL28 | Q96HX1 | TMEM50B |  |

**Supplementary table 5** Predicted genes up regulated in sporadic basal cancers, targeted by 3 or more miRNAs

| **6 mirs** | FDXR | SF1 | CACNA1S | EHMT1 | HMOX2 | NCDN | PRPF39 | STK4 |
| --- | --- | --- | --- | --- | --- | --- | --- | --- |
| KCMF1 | FOXN4 | SHANK2 | CAD | EIF1B | HMX3 | NCOR2 | PSIP1 | STOML1 |
| PHF21A | FOXP1 | SLC23A2 | CALML5 | EIF2C1 | HOXB2 | NFASC | PSORS1C2 | STYXL1 |
| TNRC6B | FRAS1 | SLC25A39 | CAMK2B | ELAVL2 | HPBP1 | NFATC3 | PTEN | SUOX |
| TNXB | FSCN1 | SLC5A3 | CAMK2D | EMILIN1 | HPS1 | NKX2-5 | PTP4A1 | SUV420H2 |
| **5 mirs** | GAL3ST1 | SORBS1 | CAMSAP1 | ENAH | HRB | NLGN2 | PTP4A3 | SVEP1 |
| C2 | GAMT | SP1 | CASK | ENPP7 | HSD11B2 | NM_001080452.1 | PTPN21 | TACC2 |
| C9orf86 | GLI3 | ST14 | CASKIN2 | EPS15 | HSFY2 | NM_022372.3 | PTPRA | TAF12 |
| CAPNS1 | GPR175 | SYTL1 | CASP8 | ESR2 | ICAM4 | NME6 | PTPRB | TAOK2 |
| CCDC114 | GPRC5C | TFAP2A | CCDC106 | ESRRG | IDH3G | NMNAT1 | PTPRS | TBCC |
| CD72 | GPRIN2 | TIMP1 | CCDC17 | ETV5 | IGLV1-51 | NMNAT2 | Q5JQD4 | tcag7.1228 |
| CLSPN | HMGA2 | TLE4 | CCDC88 | EVI5 | IGSF3 | NMT1 | Q5T8I0 | TCF7L1 |
| DLG2 | HS2ST1 | TMEM59 | CCL25 | EXOC3L2 | IHPK2 | NOSIP | Q7Z2F6 | TEX264 |
| DLGAP2 | HTR3B | TMEM67 | CCND1 | FAAH | IL27 | NOTCH4 | RAP1B | TH |
| DPYSL2 | IDH2 | TRIM14 | CD300C | FAM46B | INCENP | NOVA1 | RARA | THOP1 |
| EEFSEC | IL11RA | TRIM47 | CD34 | FAM70B | ISCA1 | NPC1 | RASGEF1C | TMED5 |
| FBXO33 | IQGAP3 | TUBGCP5 | CDC2L5 | FANCG | JHDM1D | NPHS1 | RBM34 | TMED9 |
| GRIK2 | ITGB2 | UROS | CDH23 | FBRS | JMJD2A | NR2C2 | RBM4 | TMEM16G |
| HNRNPU | ITGB4 | WDR45 | CDRT1 | FBXL8 | JUND | NRIP1 | RBM4B | TMEM16J |
| KCNMA1 | ITGB4BP | WDR86 | CEECAM1 | FBXO30 | KCNF1 | NRP1 | RBM9 | TMEM39B |
| KLK14 | KLF12 | WDR90 | CENPL | FBXO44 | KCNH6 | NTN2L | RBMS3 | TMEM41A |
| LEPRE1 | KRBA1 | YPEL3 | CENTG3 | FBXW11 | KCTD15 | NTNG2 | RBMX2 | TMEM54 |
| MLL2 | LACE1 | **3 mirs** | CFD | FEV | KCTD8 | NTRK3 | RBP5 | TMEM79 |
| MUC1 | LRRC42 | A1A5D9 | CHD1 | FGF7 | KIF9 | NUAK1 | RC3H1 | TNC |
| NFAT5 | LRRC45 | ABCC10 | CHD5 | FHL1 | KLHDC6 | NUBP2 | REPS2 | TNFRSF4 |
| NFKBIL1 | LRRTM3 | ABCF2 | CHST2 | FHOD1 | KLK8 | NUDT4 | RHCG | TP53 |
| PTGDS | LTK | ACHE | CHST8 | FIGN | KPNA6 | NUDT5 | RHOBTB1 | TPCN2 |
| RIMS3 | LYPLA2 | ACVR1C | CIB2 | FKHL18 | KRT81 | NUFIP2 | RHOT1 | TPI1 |
| SLC9A3R2 | MAD2L2 | ADAMTS6 | CLCN5 | FOXN3 | KRTAP5-4 | NUP62CL | RND2 | TRADD |
| SPTB | MAF | ADAMTSL1 | CLN3 | FRS2 | LBP | NXF1 | RNF125 | TRAF3IP2 |
| STUB1 | MAPK12 | ADCK1 | CLU | FRYL | LCAT | OFD1 | RNF139 | TRGC1 |
| TPO | MCF2L | AEBP2 | CNOT4 | FUS | LCE1B | ONECUT2 | RRAS | TRIM15 |
| TRPS1 | MDGA1 | AGBL4 | CNTFR | FXYD7 | LEPR | OPA3 | RSL1D1 | TSEN54 |
| WNT3 | MED25 | AGXT | COMP | G6PC | LHFP | OR10X1 | RTN2 | TSGA10IP |
| ZFHX4 | MTHFD1L | AHI1 | COQ4 | GATA1 | LILRB2 | OR2AE1 | RUNDC2A | TSPAN14 |
| **4 mirs** | MUSTN1 | AIM1L | CORO1C | GBGT1 | LIN54 | OR8K3 | RUVBL1 | TTBK1 |
| AAK1 | MXD3 | AKAP5 | CPSF3L | GCN5L2 | LIN7A | OSBP2 | S100A3 | TTF1 |
| AFF3 | NBPF5 | AKNA | CREBBP | GDAP1L1 | LMAN2 | OSBPL2 | SCAMP4 | TULP1 |
| ARF5 | NFIB | AKR1CL2 | CREBZF | GDF5 | LMX1B | PALM2 | SCG2 | TXNRD2 |
| ARHGAP19 | NFKBIL2 | ALKBH5 | CRKL | GFI1B | LRRC29 | PAQR3 | SCLY | UBAC1 |
| ATXN1 | NKD2 | ALOX12P2 | CRTC1 | GHRHR | LRRC48 | PCDH1 | SCN8A | UBE2I |
| BAD | NOG | ALS2CR7 | CSF2RA | GLIS3 | LRRC61 | PCDH10 | SCNN1B | UNC45A |
| BRUNOL5 | NP_659429.4 | ANKRD24 | CSMD2 | GLT8D4 | LSM10 | PCDH11X | SDCCAG8 | USP11 |
| BRWD1 | NPHP4 | ANKRD52 | CSNK1G1 | GPI | LSM12 | PCDH11Y | SEC14L1 | USP27X |
| C14orf121 | NPPB | AOF2 | CXorf34 | GPM6A | LUC7L | PCGF2 | SEC24C | USP4 |
| CACNA1C | NRBP1 | APOA1BP | CYB5A | GPM6B | LY6D | PCNXL3 | SENP3 | VAMP2 |
| CAPN13 | OBSCN | APRT | CYFIP2 | GPR45 | LY6G5B | PDLIM4 | SERPING1 | VASN |
| CASKIN1 | OLFM1 | ARHGEF1 | CYP2W1 | GPS1 | LYNX1 | PEX16 | SFRS1 | VCPIP1 |
| CC2D1A | PHF15 | ARHGEF9 | CYP4F8 | GPSM1 | LZTR2 | PEX6 | SG223 | VTI1A |
| CCDC130 | PHF8 | ARMC6 | CYP8B1 | GPX2 | MAP4K2 | PFDN4 | SH2B1 | WDR82 |
| CCDC95 | PLCB1 | ARPC4 | D2HGDH | GRAMD1A | MARCKS | PHKB | SH3PXD2A | WWC3 |
| CCM2 | PNPLA7 | ASB1 | DAP3 | GREM2 | MARK2 | PIGF | SLC22A7 | XIAP |
| CDC16 | PPP1R13L | ASXL1 | DCI | GRHL3 | MARVELD2 | PISD | SLC24A6 | XYLT1 |
| CDC42EP1 | PRKCA | ASZ1 | DCX | GRM1 | MAZ | PITPNM1 | SLC27A1 | YIPF3 |
| CLASP1 | PRKCSH | ATP1A3 | DDB2 | GTF2E1 | MED1 | PLA2G2E | SLC2A4RG | YTHDF1 |
| CNOT3 | PRPH | ATP6V1B1 | DDOST | GUCA1A | MED28 | PLA2G4B | SLC30A4 | ZBP1 |
| COL5A1 | PRR6 | ATP8A2 | DDX24 | GUCY2D | MEOX1 | PLAUR | SLC35A2 | ZBTB38 |
| CPT1B | PTOV1 | ATP9A | DEPDC5 | GYLTL1B | MESP2 | PLEKHJ1 | SMPDL3B | ZDHHC5 |
| DAB2IP | PTPN11 | ATPAF2 | DFNB31 | H2AFB2 | MGA | PLK1 | SMTNL2 | ZFAND5 |
| DEGS2 | PURB | B4GALNT3 | DLGAP1 | H2AFB3 | MIA2 | PLSCR3 | SND1 | ZFX |
| DES | PVRL1 | BACH2 | DLGAP3 | H3F3B | MICALL2 | PLXNB1 | SNTB2 | ZFYVE28 |
| DNAI2 | Q6ZRB0 | BAIAP2 | DMKN | hCG_2000329 | MKLN1 | POLN | SNX21 | ZMYND10 |
| DNAJB5 | RABGAP1L | BAZ2A | DNAJC11 | HCLS1 | MOB2 | POLR1E | SOCS6 | ZNF148 |
| DOT1L | RALGPS1 | BCAN | DRD1IP | HES6 | MON1B | POR | SOX11 | ZNF226 |
| DPM2 | RASAL2 | BCAT2 | DRD2 | HFE | MOV10L1 | PPAPDC3 | SP140 | ZNF365 |
| DTNB | RAX | BCL11B | DTX1 | HGS | MRPL12 | PPP1CB | SPATA2L | ZNF454 |
| EMID1 | RBM39 | BCL2L2 | DUSP2 | HIF3A | MRPL43 | PPP1R3E | SPNS3 | ZNF614 |
| ENSA | RGS12 | BMP1 | DYM | HIPK2 | MSI1 | PPP1R9B | SPTBN2 | ZYX |
| EXOC3 | RPS6KB2 | BMPR2 | DYNC2LI1 | HIST1H3H | MTF1 | PPP2R2C | SPTBN4 |  |
| FAM3A | RUSC2 | BNC2 | DYNLRB1 | HIVEP2 | MTMR1 | PPP2R5E | SRP9 |  |
| FAM60A | SAT1 | BRCA1 | EEF2K | HLF | MUC20 | PPRC1 | SRPR |  |
| FBXO41 | SCN9A | BRD4 | EFEMP2 | HM13 | MYH7B | PRDM14 | SSH1 |  |
| FCN3 | SENP1 | BTD | EGR3 | HMGCL | MYO7B | PRKAB2 | ST3GAL2 |  |
| FCRLB | SETD7 | BTRC | EGR4 | HMGN2 | MYT1L | PRNPIP | STARD10 |  |

**Supplementary table 6** Subset of predicted target mRNAs regulated by RISC mediated cleavage in BRCA1 and sporadic basal cancers

| **Predicted targets** | **miRNAs** | **adjusted p-value** | **Fold-Change (BRCA1 vs Basal)** |
| --- | --- | --- | --- |
| CRTAC1 | mir-10a, 149, 660 | 0.0004 | 1.87 |
| PTGDS | mir-10a, 29*, 132* | 0.0303 | 1.60 |
| LRP2 | mir-148b, 190b, 374b | 0.0311 | 1.50 |
| PLA2G5 | mir-148b, 454*, 590-3p | 0.0378 | 1.24 |
| LRMP | mir-29*, 374b, 660 | 0.0022 | 1.22 |
| FAT3 | mir-218, 454*, 590-3p | 0.0077 | 1.20 |
| ZBP1 | mir-576-3p, 198, 30c-1* | 0.0037 | -1.30 |
| TMEM16J | mir-509-5p, 129-3p, 198 | 0.0452 | -1.19 |
