# Supplementary figures and images for: Comparative microRNA profiling of sporadic and BRCA1 associated basal-like breast cancers
Source: BMC Cancer. 2015 Jul 8;15:506. doi: 10.1186/s12885-015-1522-4 (PMC4494690; doi:10.1186/s12885-015-1522-4)

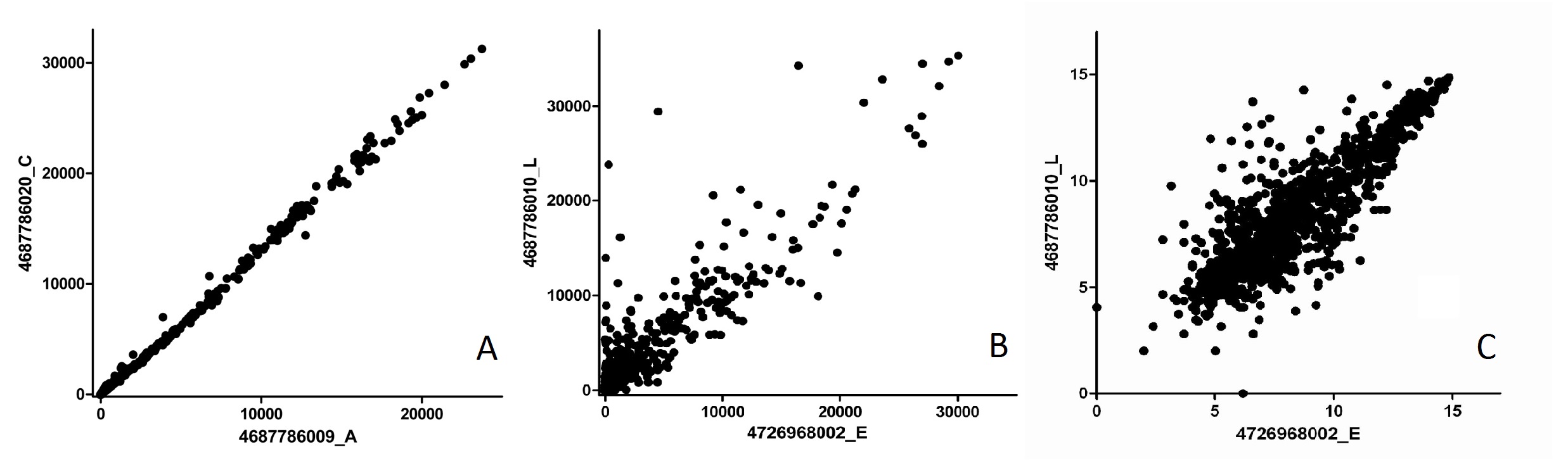

Supplement: Additional file 2: Figure S1. — Correlation of control miRNA profiles across beadchips. The probe intensities for control samples were correlated across the 6 Illumina beadchips hybridised on 2 separate runs (Run 1: 1 beadchip with 11 samples, Run 2: 5 beadchips with 58 samples). Excellent correlations for raw miRNA probe intensities were obtained between control samples across the five beadchips hybridised on the same run (Run 2, Spearman r = 0.9631–0.9906, all p < 0.001) (Additional file 2: Figure S1a). For control samples hybridised on different runs, a weaker correlation was observed (Spearman r = 0.8139, p < 0.001) (Additional file 2: Figure S1b). Occasional probes were observed to have a much stronger signal in the smaller run 1 (4687786010_L) compared to run 2 (4726968002_E). A small improvement in the correlation between these two samples was observed following quantile normalisation and log 2 transformation (Pearson r = 0.8538, p < 0.001) (Additional file 2: Figure S1c). Additional file 2: Figure S1a. Scatterplot of raw miRNA probe intensities from control samples across 2 beadchips hybridised on the same run (Spearman r = 0.9906, p < 0.001); Additional file 2: Figure S1b. Scatterplot of raw miRNA probe intensities from control samples hybridised on separate runs (Spearman r = 0.8139, p < 0.001); Additional file 2: Figure S1c. Scatterplot of probe intensities of control samples (from Additional file 2: Figure S1b), hybridised on separate runs following quantile normalisation and log 2 transformation (Pearson r = 0.8538, p < 0.001). [file 12885_2015_1522_MOESM2_ESM.jpeg]

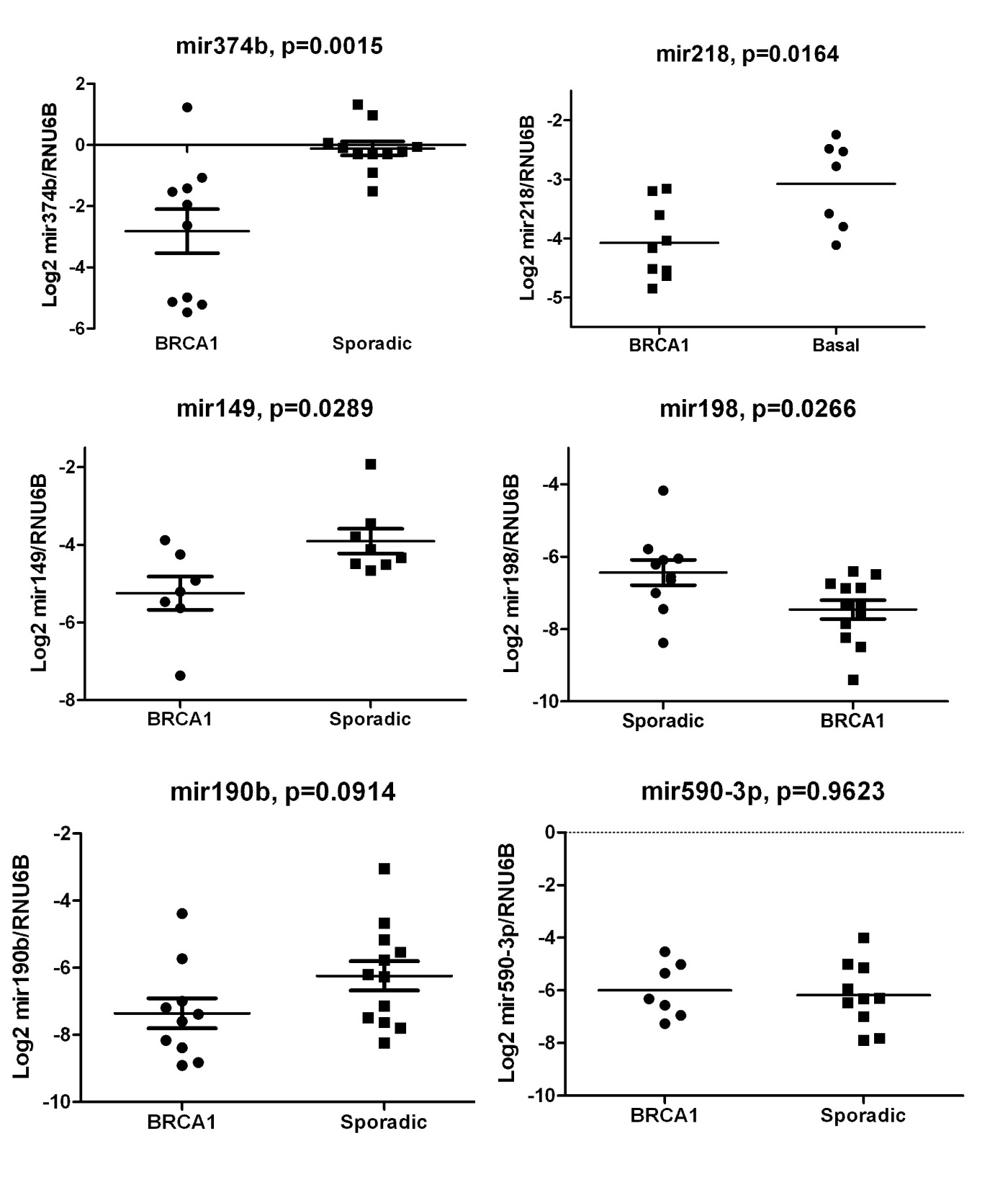

Supplement: Additional file 3: Figure S2. — RT-PCR validation of mir-374b, mir-149, mir-218 and mir-198 in discriminating between BRCA1 and sporadic basal breast cancers (all p < 0.05). A trend was seen for mir-190b although this was not statistically significant (p = 0.0914). No differences in the expression of mir-590-3p were observed (p = 0.9623). [file 12885_2015_1522_MOESM3_ESM.jpeg]

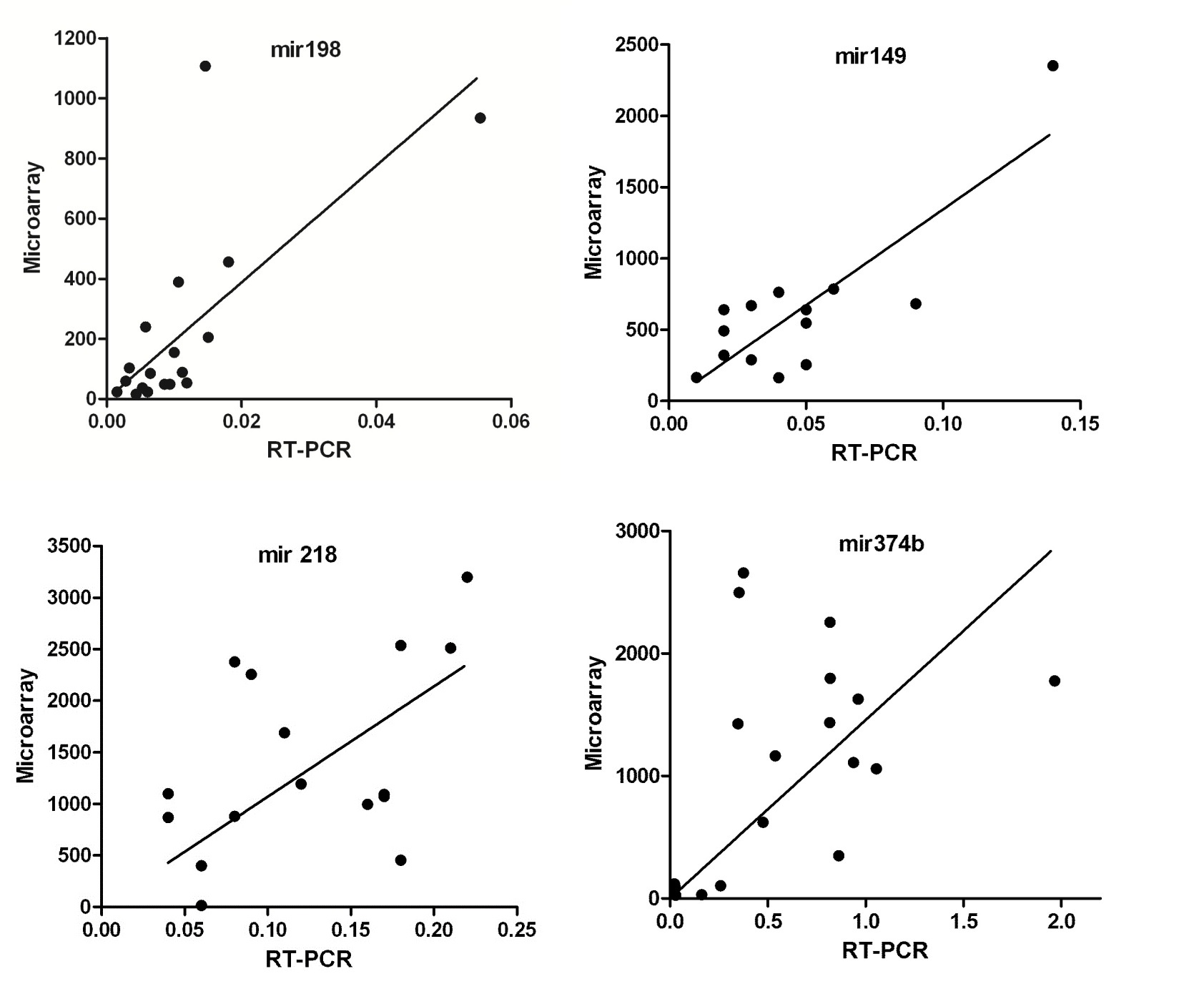

Supplement: Additional file 4: Figure S3. — Correlation between microarray and RT-PCR (relative to RNU6B) measurements for mir-198 (r = 0.672, p = 0.0023), mir-149 (r = 0.567, p = 0.0344), mir-218 (r = 0.521, p = 0.0385) and mir-374b (r = 0.503, p = 0.0335). [file 12885_2015_1522_MOESM4_ESM.jpeg]

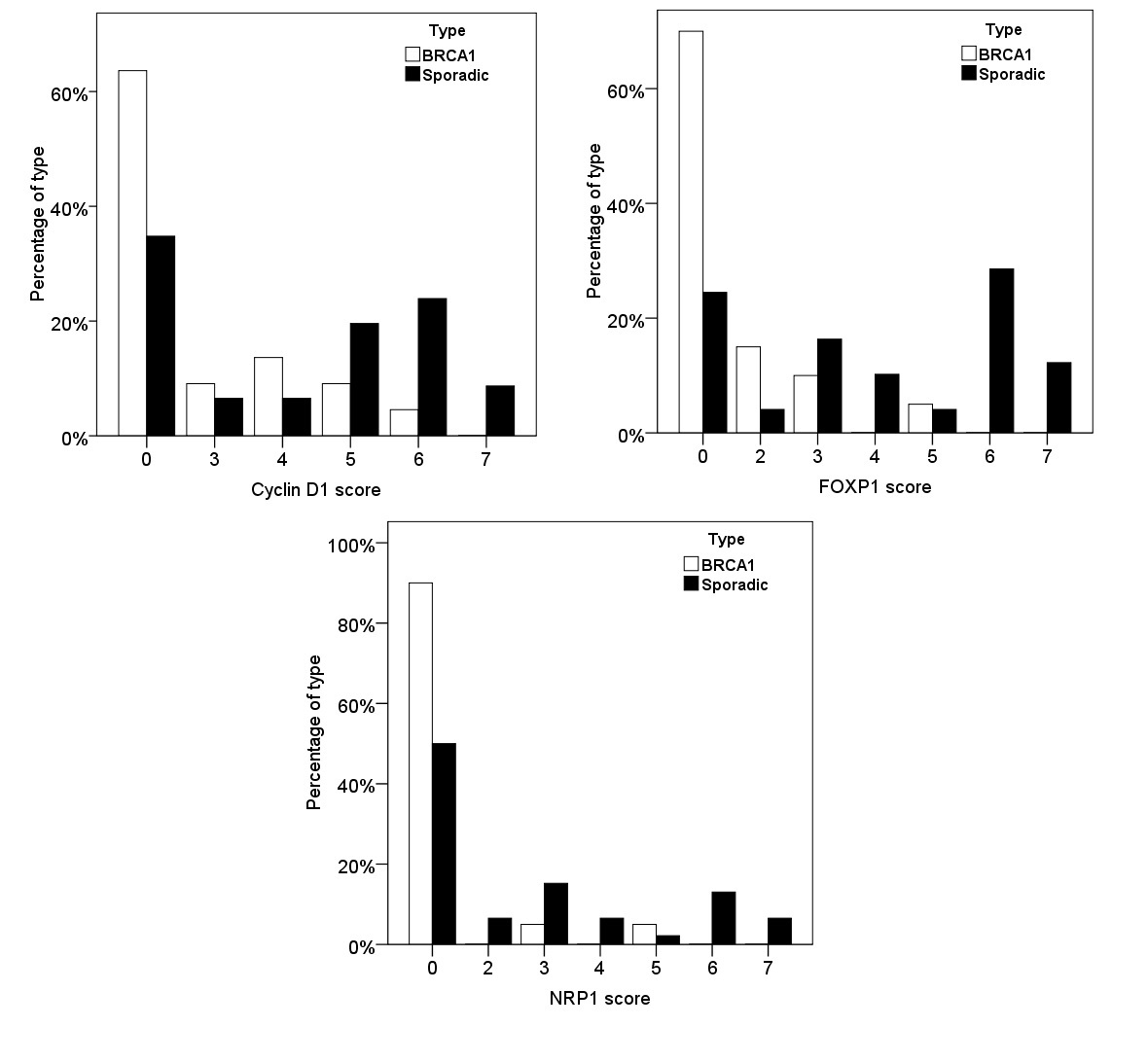

Supplement: Additional file 5: Figure S4. — Distribution of scores in BRCA1 and sporadic basal type cancers for cyclin D1 (p = 0.004), FOXP1 (p < 0.001) and NRP1 (p = 0.003). [file 12885_2015_1522_MOESM5_ESM.jpeg]
